# ApoBDs: a paradigm shift from cellular debris to therapeutic vehicles

**DOI:** 10.3389/fendo.2025.1626796

**Published:** 2025-07-17

**Authors:** Zixuan Xiong, Fen Xiao, Yuanyuan Sun, Hankun Su, Di Liu, Boya Tang, Wenyan Jian, Tianli Yang, Jing Zhao, Yanping Li, Hui Li

**Affiliations:** ^1^ Department of Reproductive Medicine, Xiangya Hospital, Central South University, Changsha, Hunan, China; ^2^ Clinical Research Center for Women’s Reproductive Health in Hunan Province, Changsha, Hunan, China; ^3^ Department of Metabolism and Endocrinology, The Second Xiangya Hospital of Central South University, Changsha, Hunan, China; ^4^ Department of Fetal Medicine, Xiangya Hospital, Central South University, Changsha, Hunan, China

**Keywords:** apoptotic bodies, apoptotic vesicles, tissue regeneration, therapeutics, diagnosis

## Abstract

Apoptosis, a genetically programmed cell death process, is essential for maintaining tissue homeostasis. Apoptotic vesicles (ApoVs), membrane-bound vesicles generated during apoptosis and once considered mere cellular debris, can be classified into apoptotic bodies (ApoBDs), microvesicles, and apoptotic extracellular vesicles (ApoEVs) based on their grain size. These vesicles, packed with bioactive molecules, not only drive tumor growth and metastasis, but also contribute to tissue and organ repair. This review focus on the origins, formation mechanisms, and dual functions of ApoBDs across various diseases, highlighting their paradoxical nature as both disease promoters and therapeutic allies. It further explores the application prospects and clinical practice of ApoBDs in cancer treatment, immune modulation, and tissue regeneration. Additionally, we provide a comprehensive perspective on the transformative potential of ApoBDs in modern medicine, while outlining current challenges and future directions for ongoing research and clinical application.

## 1 Apoptosis

Apoptosis is a genetically determined mode of programmed cell death (1). It occurs throughout the development and aging of all cells and plays a vital role in maintaining cellular homeostasis within tissues and defending against internal and external insults. This genetically determined form of programmed cell death is remarkable in that it can remove residual cellular components from adjacent tissues without triggering an inflammatory response (2, 3). Apoptosis critically regulates multiple physiological processes, including normal cell renewal, normal development and function of the immune system, hormone-dependent atrophy, embryonic development, and chemically induced cell death. Dysregulated apoptosis may contribute to various pathologies such as neurodegeneration, tissue damage, autoimmune disorders and various types of cancer (4).

## 2 The process of apoptosis

**Figure 1:**
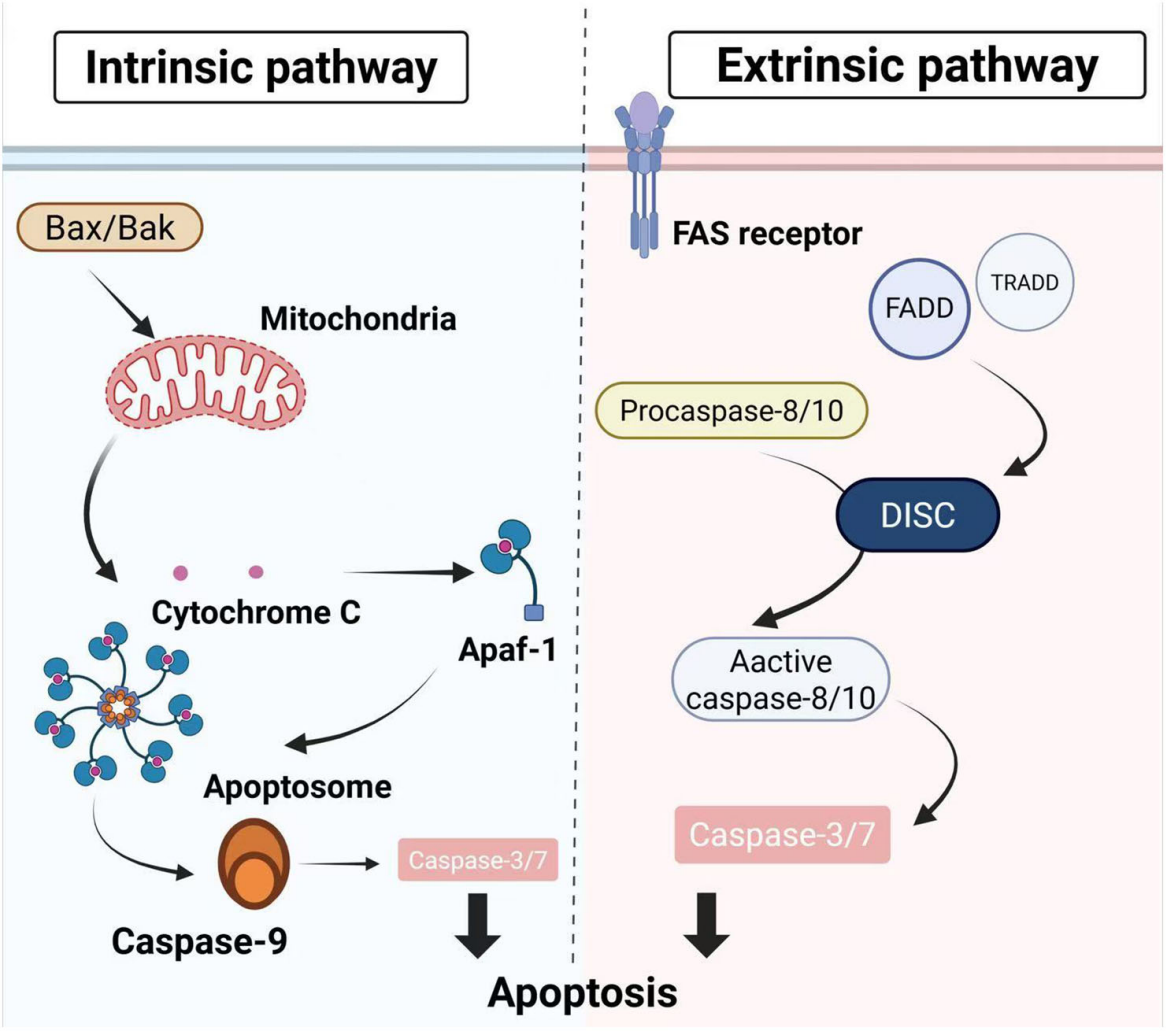
The process of cell apoptosis. There are two principal apoptotic pathways. The intrinsic pathway: cells respond to stress by prompting Bax/Bak oligomerization and mitochondrial outer membrane permeabilization and the release of factors such as cytochrome c. These factors facilitate assembly of the Apaf-1-caspase-9-apoptosome, leading to caspase-3/7 activation. The extrinsic pathway: it is initiated by ligand binding to death receptors (e.g., FAS) of the TNF receptor superfamily, which recruits FADD and triggers formation of the death-inducing signaling complex (DISC), activation of initiator caspases-8 and -10, and subsequent activation of caspase-3/7 to drive apoptosis.

There are two ways of apoptosis shown in **Figure 1**, intrinsic and extrinsic pathways, both of which converge on activation of caspase-3/7 to complete the whole process of apoptosis (5–7). The intrinsic pathway is mainly regulated by BCL-2 family proteins (8, 9), which comprise three structurally and functionally related subgroups: (i) BH3 protein, which senses cellular stress and initiate the apoptotic cascade; (ii) the pro-apoptotic effectors Bax and Bak, which oligomerize in the mitochondrial outer membrane to permeabilize it and release intermembrane factors that activate downstream caspases; and (iii) anti-apoptotic members such as BCL-2 itself (10, 11). Key pro-apoptotic proteins released into the intermembrane space include cytochrome c, Smac/DIABLO, and the serine protease HtrA2/Omi, with cytochrome c binding apoptotic protease activating factor-1 (Apaf-1) to form the apoptosome and mediate caspase-9 activation (12, 13). Extrinsic pathways are mainly mediated by extrinsic signals through receptors that transmit signals to initiate apoptosis, involving death receptors as members of the tumor necrosis factors (TNF) receptor superfamily (14, 15). Members of the TNF receptor family share a conserved intracellular domain called the “Death Domain” (16). Ligand binding induces assembly of the death-inducing signaling complex (DISC), which recruits and activates caspase-8 or caspase-10, thereby initiating the apoptotic program (17, 18).

## 3 The generation mechanism of ApoBDs

EVs are membrane‐derived structures that play critical roles in intercellular communication, immune regulation, cell proliferation and differentiation, development, and regeneration (6, 19, 20). In the late stages of apoptosis, cells undergo a series of regulated morphological changes that give rise to various apoptotic vesicles collectively referred to as EVs, of which apoptotic bodies (ApoBDs) represent the principal component (21). ApoBDs are membrane‐bound vesicles released during the terminal phase of programmed cell death; they are the largest among all ApoVs subtypes, with diameters of approximately 1–5 μm, compared to microvesicles (50–1,000 nm) and exosomes (30–100 nm) (10) (**Box 1**). Typical ApoBDs contain components such as DNA, mRNA, miRNA, lipids, and proteins; however, the precise cargo composition and surface markers of tumor cell–derived ApoBDs remain incompletely characterized (22).

Box 1 **The definition of the three major classes of membrane-bound extracellular vesicles (EVs).**

Apoptotic bodies (ApoBDs)

Apoptotic bodies are the largest subclass of extracellular vesicles (1–5 μm) generated during the morphological disassembly of apoptotic cells.

Microvesicles

Microvesicles are 50–1000 nm vesicles shed from the plasma membrane of apoptotic cells.

Exosomes

Exosomes are the smallest (30–100 nm) and most thoroughly studied type of EVs.

**Figure 2:**
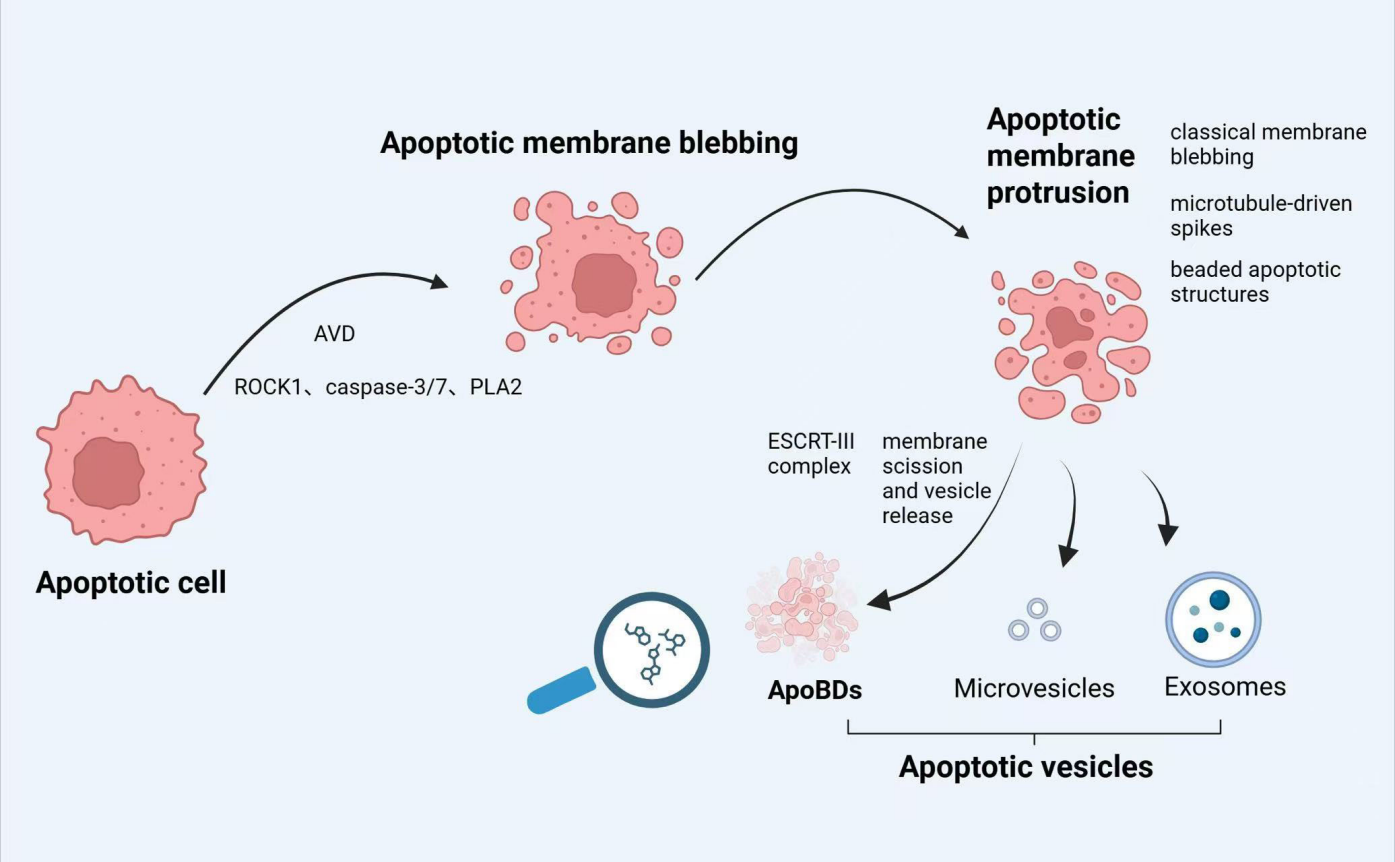
The generation of ApoBDs. The disassembly of apoptotic cells into ApoBDs occurs in three sequential stages. First, executioner caspase-3/7 activation of ROCK1, together with PLA_2_-mediated alterations in transmembrane pressure, drives rapid cell shrinkage and the formation of spherical membrane blebs on the apoptotic cell surface. Second, it involves apoptotic membrane protrusions. Different apoptotic cells exhibit distinct membrane deformation patterns, such as membrane blebbing, others manifest microtubule-driven spikes or beaded apoptotic structures. Third, apoptotic cells undergo fragmentation: ESCRT-III complex–mediated neck constriction of membrane protrusions results in scission and release of vesicles containing organelles and nuclear fragments, which mature into ApoBDs.

The formation of ApoBDs is a “highly regulated” process comprising three precisely orchestrated morphological stages (10) (**Figure 2**). In the first stage, apoptotic cells develop spherical membrane blebs on their surface. Executioner caspases, primarily caspase-3/7, cleave and activate Rho-associated kinase 1 (ROCK1) (rather than PAK2 or LIMK), whereupon ROCK1 phosphorylates myosin light chain (MLC) to drive actin–myosin contraction (23–25). Simultaneously, phospholipase A_2_ (PLA_2_) modulates the intracellular–extracellular hydrostatic pressure imbalance, promoting rapid cell shrinkage and membrane blebbing (26, 27). Early apoptotic volume decrease (AVD) further primes bleb formation by inducing cytoskeletal reorganization and ion channel regulation, leading to cell contraction and nuclear envelope rupture and thereby enhancing blebbing efficiency (28).

The second step involves the formation of apoptotic membrane protrusions. During this phase, different cell types exhibit distinct membrane deformation patterns: although most cells undergo classical membrane blebbing, others display microtubule‐driven spikes or beaded apoptotic structures. For example, neurons and certain epithelial cells form microtubule spikes, whereas apoptotic THP-1 cells and primary human neutrophils develop beaded membrane structures. Notably, apoptotic microvesiculation represents the most efficient mechanism for generating ApoBDs, yielding approximately 10–20 vesicles per cell (22).

The third step involves the fragmentation of apoptotic cells to generate a spectrum of apoptotic vesicles, including ApoBDs (29). As the neck of each budding vesicle constricts further, the ESCRT-III complex (e.g., CHMP4B) is recruited to the plasma membrane to mediate membrane scission and vesicle release (30).Through continued contraction and fusion of these apoptotic membrane structures, vesicles of varying sizes containing organellar fragments and nuclear debris ultimately mature into ApoBDs, which are subsequently released into the extracellular milieu via exocytosis or membrane rupture. Under homeostatic conditions, released ApoBDs present a coordinated array of “Find-Me” and “Eat-Me” signals to attract and facilitate uptake by macrophages or other phagocyte (31, 32). Typical “Find-Me” signals include ATP/UDP released through PANX1 channels, lysophosphatidylcholine (LPC), sphingosine-1-phosphate (S1P), and soluble C‐X3‐C motif chemokine ligand 1 (CX3CL1), which together establish chemotactic gradients that draw macrophages to the site of apoptosis (33, 34). Thereafter, surface “Eat-Me” signals on apoptotic membranes directly facilitate efferocytosis: caspase-mediated cleavage of Xkr8 activates an ATP-dependent P4-ATPase flippase (P4-ATP flippase), resulting in the externalization of phosphatidylserine (PS), which is recognized by phagocytic receptors such as Tim-4, BAI1, and αvβ3 integrin, or via bridging by MFG-E8 to αvβ3 integrin (35). In addition, calreticulin translocation to the cell surface binds to LRP1 receptors on macrophages, synergizing with PS to enhance phagocytic efficiency. SLAMF7 and Fc region exposure have also been identified as “Eat-Me” signal (33). Through this highly orchestrated series of events, ApoBDs are not only efficiently cleared but also deliver their cargo of miRNAs, proteins, and metabolites to neighboring cells, thereby executing immunoregulatory, regenerative, pro-regeneration/repair or pathological progression.

## 4 The function of ApoBDs in diseases

### 4.1 The dual role of ApoBDs in tumors

Malignant tumor cells typically exhibit a high proliferative capacity yet, paradoxically, also display elevated apoptosis rates; for example, both the indolent basal cell carcinoma (BCC) and the aggressively growing glioblastoma multiforme (GBM) manifest this phenotype (36, 37). A wealth of evidence suggests that high proliferation and high apoptosis are not strictly antagonistic but rather may interact via ApoBDs and related mediators to jointly influence tumor progression or suppression.

#### 4.1.1 Pro-tumorigenic effects

Although tumor-derived apoptotic vesicles (including ApoBDs) are often not meticulously categorized in most studies, they play critical roles in post‐treatment tumor cell proliferation and metastatic dissemination (38, 39). For example, in highly aggressive malignancies such as GBM, apoptotic tumor cells frequently intermingle with surviving counterparts. Apoptotic vesicles released from dying cells promote proliferation, migration, and therapy resistance in adjacent surviving tumor cells through multiple signaling pathways, potentially involving RBM11-mediated splicing regulation (40). RBM11 is markedly overexpressed in tumor cells following therapy and dissociates from dying cells within ApoBDs. Upon co‐uptake into recipient cells with apoptotic vesicles, RBM11 re‐splices MDM4 and cyclin D1 transcripts to generate isoforms with enhanced oncogenic potential, thereby augmenting the proliferative and resistant phenotype of post‐treatment tumor cells (40). Similarly, another study by Huang et al. (41) investigated how chemotherapy‐induced apoptosis of tumor cells influences the regrowth of surviving cancer cells and identified a previously unrecognized, caspase‐dependent regenerative mechanism. They demonstrated that apoptosis of tumor cells induced by chemotherapy or radiotherapy, partially mediated by caspase activation, paradoxically stimulates repopulation of the residual tumor cell population. Importantly, pharmacological inhibition of caspases effectively disrupts this “apoptosis–regeneration” cycle, thereby enhancing therapeutic efficacy. These results highlight a novel strategy for improving cancer radiotherapy outcomes by targeting caspase activity to prevent tumor repopulation following treatment.

Furthermore, the PS on the surface of ApoBDs can recruit the tumor-associated ligand GAS6 to engage the AXL receptor (42–44). AXL, a receptor tyrosine kinase overexpressed across a variety of malignancies, is known to drive cancer cell migration and therapeutic resistance upon activation (42). Thus, the PS–GAS6–AXL signaling axis represents a critical promoter of tumor invasion and metastasis, and targeting this axis may effectively suppress tumor progression (42, 44).

Furthermore, the role of tumor-associated macrophages (TAMs) in promoting tumor growth and progression is closely linked to apoptosis. By engulfing ApoBDs, TAMs not only contribute to angiogenesis and matrix remodeling but also suppress antitumor immunity, thereby facilitating tumor expansion (45, 46). In aggressive non-Hodgkin lymphoma (NHL), apoptosis accelerates the recruitment of TAMs to tumor sites and promotes both angiogenesis and metastatic dissemination (46, 47). In prostate cancer specimens, the phagocytic activity of TAMs toward ApoBDs is significantly elevated compared with adjacent normal prostate tissue (48). Moreover, the number of ApoBDs correlates positively with Gleason grade. This suggests that increased apoptosis is a feature of higher malignancy and may serve as a histological marker for diagnosing high-grade prostate cancer in biopsy specimens (49, 50). Although the precise mechanisms remain to be elucidated, these observations support a pro-tumor role for apoptosis across diverse cancer types.

#### 4.1.2 Anti-tumorigenic effects

In fact, tumor cell–derived apoptotic vesicles have demonstrated unique advantages in cancer immunotherapy owing to their intrinsic capacity to serve as antigen carriers (51). Among these, ApoBDs, owing to their intrinsic biogenetic mechanisms and cargo characteristics, synergize effectively with dendritic cells (DCs)’ superior capacities for antigen uptake, processing, and presentation. The DCs represent a critical reagent in tumor immunotherapy (52, 53). Studies have shown that, compared with tumor lysates and free RNA, DCs internalize and process tumor cell–derived ApoBDs more efficiently, thereby eliciting a more potent antitumor immune response (54). Although apoptotic vesicles derived from murine B16 melanoma cells exhibit higher levels of fibrin and thrombin generation, resulting in significantly enhanced procoagulant activity, this may contribute to thrombus formation in cancer patients (55). However, ApoBDs exhibit the highest anti-tumor activity among the EVs derived from murine B16 melanoma cells, including exosomes, microvesicles, and ApoBDs. This is primarily attributed to their enrichment in immunologically active components, such as HMGB1 and calreticulin translocation, released during immunogenic cell death (56). K. Zhang et al. optimized triple-negative breast cancer (TNBC)-derived ApoBDs via chemical induction and extrusion, then loaded them with saponin cytotoxin and anti-Twist siRNA. These drug-loaded ApoBDs exhibited excellent antitumor efficacy in an orthotopic TNBC metastasis model, significantly inhibiting both tumor growth and pulmonary dissemination (57).

Moreover, exogenous ApoBDs have demonstrated potential in overcoming tumor drug resistance. For instance, the “bystander effect” mediated by ApoBDs can enhance the intratumoral penetration and efficacy of chemotherapeutic agents (58). It has been reported that camptothecin (CPT) can induce the generation of ApoBDs from tumor cells under normoxic conditions, while the hypoxia-activated prodrug PR104A can be transported via these ApoBDs to adjacent tumor cells, thereby improving the efficiency of chemotherapy (59). In addition, tumor cell-derived ApoBDs can serve as natural carriers for antitumor vaccines, as the tumor-specific antigens they carry are capable of eliciting robust antitumor immune responses (60, 61).

In summary, tumor cell-derived ApoBDs can exert dual roles under different microenvironmental and therapeutic contexts. They may promote tumor proliferation, invasion, angiogenesis, and immune evasion through various signaling axes, while also serving as efficient antigen carriers to enhance the efficacy of tumor immunotherapy. Elucidating the underlying molecular mechanisms in greater depth will facilitate the suppression of their tumor-promoting functions and maximize their potential in anti-tumor therapeutic applications.

### 4.2 Role of ApoBDs in tissue repair

ApoBDs are no longer merely regarded as “fragments” of apoptotic cells, but rather as “messengers” that actively promote tissue repair. By being phagocytized by various types of recipient cells, ApoBDs initiate a cascade of signaling pathways that mediate intercellular communication. This enables them to exert broad and efficient reparative effects across multiple tissues, including bone, heart, skin, liver, and epithelium. Emerging evidence underscores the potential of ApoBDs as a versatile therapeutic platform: they not only modulate immune responses but also directly activate regenerative processes, recapitulate the reparative functions of their parent cells, and circumvent many limitations associated with conventional cell therapies. Consequently, ApoBDs represent a promising frontier in the field of precision regenerative medicine.

#### 4.2.1 Repair of skin and wound healing

In recent years, numerous studies have demonstrated that ApoBDs exhibit significant therapeutic efficacy in skin injury and chronic wound repair, primarily through the modulation of macrophage (Mφ) polarization and function. Multiple inflammatory signaling pathways act synergistically to regulate the transition of macrophages from a pro-inflammatory M1 phenotype to an anti-inflammatory M2 phenotype, thereby promoting wound healing (62). ApoBDs derived from mesenchymal stem cells (MSCs) have been shown to carry miR-21-5p, which targets and suppresses KLF6 expression, thus inducing anti-inflammatory M2 polarization (63). These M2 macrophages, upon stimulation by ApoBDs, can further enhance fibroblast proliferation and migration, accelerating wound closure, re-epithelialization, and hair follicle regeneration in murine models (64). In addition, Mao et al. reported that ApoBDs derived from adipose-derived stem cells (ADSCs) modulate macrophage inflammatory polarization via the miR-20a-5p-regulated JAK/STAT signaling pathway, thereby facilitating the repair of chronic wounds (65). Moreover, MSC-secreted TSG6 has been found to further restrict excessive macrophage activation, inhibit myofibroblast differentiation, and reduce excessive collagen deposition, significantly alleviating chronic inflammatory scarring (62, 66).

Meanwhile, ApoBDs are readily engulfed by macrophages through efferocytosis, a process that plays a critical role in the transition from the early inflammatory phase to the proliferative phase. Macrophages that phagocytose apoptotic cells not only attenuate pro-inflammatory signaling but also create a permissive environment for the initiation of tissue repair (67, 68). Moreover, active caspase-3/7 released from apoptotic cells can activate PLA_2_, leading to the production of prostaglandin E_2_ (PGE_2_), which in turn triggers the so-called “Phoenix Rising” pathway. This pathway directly promotes the proliferation of resident stem/progenitor cells and accelerates wound regeneration (69).

ApoBDs also play a pivotal role in epithelial repair via the Wnt/β-catenin pathway. In epithelial tissues, apoptosis of stem cells leads to the caspase-dependent generation of ApoBDs enriched in Wnt8a. Following engulfment by adjacent basal stem cells, these ApoBDs activate Wnt signaling, thereby stimulating stem cell proliferation to replace damaged cells and sustain both stem cell numbers and epithelial homeostasis (70). The β-catenin pathway is well-established as critical for normal hair follicle development and cycling (71). Although allogeneic stem cell transplantation has been reported to produce exogenous apoptotic extracellular vesicles that activate the Wnt/β-catenin pathway in skin and hair follicle mesenchymal stem cells, promoting wound healing and hair regeneration (72), this mechanism does not explicitly link MSC-derived ApoBDs with these effects. Therefore, based on the syntenic cognate between ApoBDs and apoptotic extracellular vesicles, future experimental validation is required to confirm whether ApoBDs perform analogous functions in the aforementioned contexts. Furthermore, human bone marrow-derived MSCs (BMSCs) ApoBDs have been shown to induce macrophage polarization toward the M2, secreting PGE_2_ and TGF-β. This fosters an immunoprivileged milieu, enhances angiogenesis, stimulates fibroblast proliferation and migration, and remodels the extracellular matrix—thereby optimizing both the structural and functional aspects of tissue repair (73).

Collectively, these studies demonstrate that ApoBDs facilitate rapid and high-quality tissue regeneration by synergistically modulating immune responses, activating signaling pathways, coupling clearance with regeneration, and remodeling the vascular extracellular matrix. These multifaceted mechanisms offer novel perspectives for enhancing skin tissue repair and wound healing.

#### 4.2.2 Repair of vascular damage

Arterial calcification is commonly observed in the early stages of arteriosclerosis and is accompanied by endothelial dysfunction and apoptosis. Recent studies have demonstrated that, in a murine model of arteriosclerosis, endothelial cell-derived ApoVs are enriched with miR-126, which can be taken up by adjacent smooth muscle cells and monocytes/macrophages. This uptake suppresses RGS16, a G protein signaling regulator, thereby enhancing the responsiveness of CXCR4 to its ligand CXCL12. This establishes a positive autoregulatory feedback loop that promotes CXCL12 secretion and CXCL12-dependent vascular protection, ultimately inhibiting the formation of calcified plaques and progression of arteriosclerosis (74). Furthermore, apoptotic vesicles generated under oxidative stress conditions create a reactive ROS-rich microenvironment that targets vascular endothelial cells. Notably, these oxidative stress-induced apoptotic vesicles (Oxi-ApoVs) exhibit superior performance in *in vitro* lumen formation assays, highlighting their potential for mimicking pathological microenvironments and precisely regulating angiogenesis (75).

Further subclassification of these functional apoptotic vesicles reveals that endothelial cell-derived ApoBDs can serve as carriers that deliver reparative signaling molecules, thereby playing a critical role in the reversal of atherosclerosis and the inhibition of vascular calcification (76, 77). Moreover, endothelial progenitor cells (EPCs) are key effector cells in vascular regeneration (78). *In vitro* studies by Hristov M et al. demonstrated that uptake of endothelial-derived ApoBDs by EPCs markedly enhances their number and differentiation maturity, directly supporting endothelial repair (79). Conversely, apoptotic bodies originating from vascular smooth muscle cells (VSMCs) act as nucleation sites for calcification, rapidly inducing calcium salt deposition within the arterial wall. Inhibition of VSMC apoptosis substantially reduces calcification, whereas promotion of apoptosis exacerbates it, highlighting the driving role of ApoBDs in vascular calcification (77, 80).

In summary, ApoBD, as a multifunctional carrier of signals and substances, enables precise delivery of various pro-angiogenic factors during endothelial injury repair, offering a novel approach for cardiovascular disease intervention. Meanwhile, ApoBDs derived from different sources and carrying distinct cargos exhibit opposing effects in promoting healthy vascular regeneration versus exacerbating pathological calcification. Therefore, a thorough elucidation of their cellular origins and carried factors, combined with targeted interventions tailored to specific pathological stages, will be crucial for harnessing ApoBDs in multi-level, multi-stage cardiovascular protection and regenerative medicine applications.

#### 4.2.3 Repair of endometrial injuries

Intrauterine adhesions (IUAs) are primarily characterized by fibrosis and adhesion formation following damage to the basal layer of the endometrium, commonly occurring in women with a history of miscarriage or delivery complicated by curettage (81). Conventional treatments for IUAs, such as traditional intrauterine devices and estrogen therapy, fail to adequately promote endometrial regeneration (82). Current mechanistic studies indicate that the synergistic interaction between the TGF-β and Wnt/β-catenin signaling pathways is a critical driver of endometrial fibrosis in IUAs (83, 84). Recent research has demonstrated that ApoBDs derived from BMSCs can inhibit TGF-β1-induced fibrosis by suppressing the Wnt/β-catenin signaling pathway (85). Mechanistically, ApoBDs may carry bioactive factors including miRNAs and proteins that downregulate β-catenin and its downstream targets, such as Cyclin D1 and c-Myc, thereby inhibiting the proliferation and myofibroblastic differentiation of human endometrial stromal cells (HESCs) (85). However, other studies have reported that MSCs engulfing ApoBDs utilize the RNF146 and miR-328-3p contained within the ApoBDs to degrade Axin1, thereby activating the Wnt/β-catenin pathway to maintain their stemness (86). The overactivation of the Wnt/β-catenin pathway further promotes fibrosis of the endometrium (87). Therefore, ApoBDs exhibit bidirectional regulation of the Wnt/β-catenin signaling pathway in different cell types. However, the underlying molecular mechanisms remain incompletely understood. Whether ApoBDs activate or inhibit this pathway may depend on their cellular origin, the target cell type, and the specific factors they carry.

In recent years, research on repairing the endometrium via stem cell transplantation has progressed rapidly; however, certain limitations still exist, prompting continued exploration of novel therapeutic strategies (88, 89). In 2021, Xin L et al. proposed an innovative treatment approach based on MSC-derived exosome therapeutic regimen, wherein ApoBDs derived from human umbilical cord mesenchymal stem cells (huMSCs) were loaded into a hydrogel and delivered *in situ* to the endometrium (90). This method was shown to induce macrophage immunomodulation, cell proliferation, and angiogenesis *in vitro* (90). This strategy holds promise for the treatment of ovarian cancer while simultaneously reducing fibrosis and promoting endometrial regeneration. Its key advantage lies in its “cell-free” nature, which circumvents the immunogenicity, potential tumorigenicity, and low transplantation efficiency associated with stem cell transplantation, while the injectable delivery minimizes tissue trauma and adapts well to irregular uterine cavity morphology (90). Therefore, ApoBDs-loaded hyaluronic acid (HA) hydrogels offer a feasible clinical treatment paradigm by enabling sustained release of ApoBDs to achieve immunomodulation and regeneration of the endometrium.

In summary, ApoBDs not only reverse endometrial fibrosis but also facilitate efficient tissue regeneration via localized controlled-release systems, providing a solid scientific basis for extracellular therapy of intrauterine adhesions.

#### 4.2.4 Repair of osteoporosis and osteoarthritis

The maintenance of bone homeostasis depends on the dynamic balance and mutual regulation between osteoclasts and osteoblasts (91). Signals released by osteoblasts can activate osteoclasts, which have a lifespan of approximately two weeks. Upon apoptosis, osteoclasts release a large number of ApoBDs. These osteoclast-derived ApoBDs can activate reverse signaling of RANKL in pre-osteoblasts, thereby initiating the PI3K/AKT/mTOR/S6K signaling cascade. This process enhances the expression of osteogenic markers and mineralization capacity, coupling bone resorption with bone formation and ultimately promoting bone remodeling (92). Meanwhile, apoptosis of BMSCs and the release of their associated ApoBDs also play a critical role in maintaining bone homeostasis. Liu et al. demonstrated that BMSC-derived ApoBDs not only sustain self-renewal and multilineage differentiation potential but also restore and enhance tissue regeneration and immunomodulatory functions, thereby improving bone homeostasis and exerting therapeutic effects on osteoporosis (86). In addition, under hypoxic conditions, ApoBDs released by BMSCs can be phagocytosed via DC-STAMP-mediated mechanisms, delivering miR-223-3p–enriched ApoBDs to pre-osteoclasts. This delivery inhibits osteoclast differentiation and bone resorption, thereby attenuating alveolar bone loss caused by periodontitis and highlighting the potential of ApoBDs in the treatment of inflammatory bone diseases such as periodontitis (93).

In inflammatory osteoarticular disorders, ApoBDs have been shown to induce the polarization of macrophages toward an anti-inflammatory phenotype and suppress inflammatory responses. Qin et al. reported that ApoBDs derived from M2 macrophages are enriched in anti-inflammatory miR-21-5p. These ApoBDs can be internalized by M1 macrophages, reprogramming them into M2, thereby suggesting the therapeutic potential of ApoBDs in alleviating articular cartilage damage and chronic inflammation (94). Interestingly, Chen et al. engineered T cell–derived ApoBDs with both immunomodulatory properties and cartilage-targeting affinity. These engineered ApoBDs were encapsulated into lubricating hydrogel microspheres to construct an injectable multifunctional microsphere system. This system not only modulates the inflammatory microenvironment and maintains cartilage homeostasis via biochemical cues but also reduces cartilage friction through physical lubrication, significantly promoting cartilage regeneration and improving outcomes in osteoarthritis treatment (95).

Collectively, these studies indicate that ApoBDs facilitate the repair of inflammatory bone injuries and osteoarticular tissues, highlighting their promising therapeutic potential in diseases such as osteoporosis.

#### 4.2.5 Repair of live fibrosis

Hepatic stellate cells (HSCs) play a central role in the progression of liver fibrosis. Upon fibrogenic stimulation, HSCs become activated and transdifferentiate into myofibroblasts that secrete procollagen alpha1 (I) (96). It is generally believed that inducing apoptosis in HSCs can suppress the progression of fibrosis (97). However, when HSCs engulf ApoBDs derived from damaged hepatocytes such as HepG2 cells, fibrosis is not inhibited but instead exacerbated. Jiang JX et al. reported that the phagocytosis of HepG2-derived ApoBDs by HSCs promotes the dissemination of survival signals via JAK1/STAT3-dependent and Akt-dependent pathways. This process also upregulates NF-κB and the anti-apoptotic proteins Mcl-1 and A1, thereby enhancing the survival of HSCs and facilitating collagen deposition (98). Recent studies further reveal that within the fibrotic liver microenvironment, ApoBDs can induce the upregulation of TGF-β1 expression and increase the synthesis of procollagen alpha1 (I) in HSCs. This effect is accompanied by the activation of NADPH oxidase and elevated reactive oxygen species (ROS) levels, which further initiate downstream signaling pathways such as PI3K and p38 MAPK. These cascades amplify the TGF-β/Smad fibrotic signaling, leading to sustained pathological extracellular matrix (ECM) accumulation and aggravated fibrosis (99).

Notably, ApoBDs not only carry cellular “debris” from apoptotic cells but also serve as signaling carriers for profibrotic factors, delivering key molecules such as hepatocyte growth factor (HGF) to HSCs, thereby further enhancing fibrogenic activity. However, only HIV-induced apoptotic bodies derived from hepatocytes have been shown to significantly activate profibrotic gene expression in HSCs and promote liver fibrosis progression, whereas ApoBDs originating from immune cells do not exhibit this effect (100, 101). Based on this specificity, recent studies have explored the use of chemically induced hepatocyte-like cells (ciHeps) to generate engineered apoptotic vesicles (ciHep-apoVs). By loading these vesicles with specific microRNAs, researchers have successfully suppressed glycolysis, PI3K/AKT/mTOR signaling, and epithelial–mesenchymal transition (EMT) pathways, thereby effectively blocking the proliferation and collagen production of activated HSCs (102). As multifunctional nanotherapeutics, these ciHep-apoVs not only demonstrate significant antifibrotic potential in liver disease but also provide a novel strategy for the engineered ApoBDs-based treatment of fibrotic diseases in other organs.

## 5 Mechanisms of ApoBDs action in diseases

**Figure 3:**
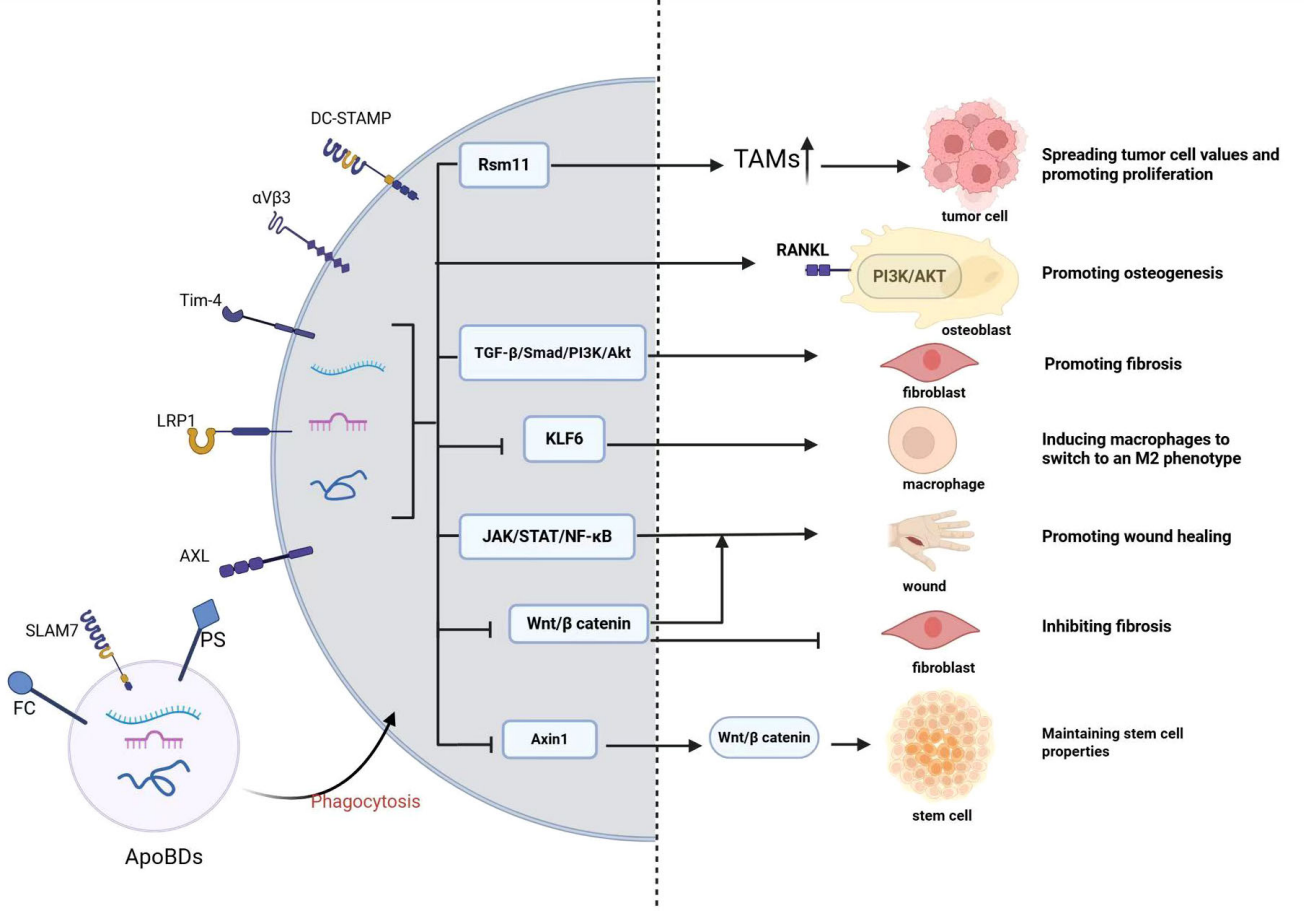
Mechanisms of ApoBDs action in diseases. ApoBDs carry various cargos such as miRNA, DNA, and proteins, which regulate the phenotype of target cells through the aforementioned pathways or synergistic mechanisms, thereby influencing disease progression or tissue repair.

ApoBDs play a complex and pivotal role in a variety of diseases, primarily functioning as efficient biological information carriers that mediate intercellular communication and regulate disease progression (**Figure 3**, **Table 1**). First, ApoBDs are enriched with bioactive components derived from their parental cells, such as miRNAs, mRNAs, and proteins (**Table 2**). Upon active uptake by adjacent or distant specific recipient cells, such as macrophages, stem cells, fibroblasts, endothelial cells, hepatic stellate cells, or tumor cells, the carried signaling molecules can directly modulate gene expression and signaling pathways in the recipient cells. This modulation may promote tissue repair and regeneration (e.g., activation of the Wnt/β-catenin pathway to enhance epithelial or bone regeneration; miR-126-mediated vascular protection; and induction of M2 macrophage polarization to suppress inflammation), or, conversely, facilitate pathological signaling (e.g., RBM11-mediated alternative splicing promoting tumor proliferation and drug resistance, or hepatocyte-derived ApoBDs activating fibrogenic pathways in hepatic stellate cells). Second, surface molecules on ApoBDs, such as exposed PS, act as “eat-me” signals to mediate their clearance by phagocytes such as macrophages and dendritic cells. This process itself triggers critical immunomodulatory and anti-inflammatory responses, as well as the release of regenerative signals (such as the “Phoenix Rising” pathway). Third, the effects of ApoBDs are highly context-dependent and source-specific, with bidirectional roles observed. In the tumor microenvironment, ApoBDs may promote tumor growth, invasion, angiogenesis, and immune evasion; yet they can also serve as potent antigen carriers to elicit robust anti-tumor immunity. In tissue repair, ApoBDs generally support regeneration; however, under specific conditions, such as those derived from vascular smooth muscle cells, they may drive pathological calcification, or, when originating from specific hepatocyte subpopulations, exacerbate fibrosis. Therefore, a comprehensive understanding of the cargo composition, cellular origin, recipient cell types, and the surrounding microenvironment of ApoBDs is essential for elucidating their disease-related mechanisms and for the development of ApoBDs-based targeted therapeutic strategies, such as engineered ApoBDs for drug delivery.

**Table 1:** The regulatory functions of ApoBDs in diseases.

Disease	Type of disease	ApoBDs-derived cells	Biological functions	Mechanism of action	References
Tumor	Glioblastoma multiforme	Glioblastoma multiforme	Promoting the proliferation and drug resistance of surviving tumor cells	Shifting the splicing of MDM4 and cyclin D1 towards expression of more oncogenic subtypes by RBM11	(40)
	Aggressive non-Hodgkin lymphoma	Aggressive non-Hodgkin lymphoma	Promoting the growth and angiogenesis of aggressive non-Hodgkin lymphoma	Promoting cancer-promoting TAM accumulation	(45–47)
	Prostate cancer	Prostate cancer cells	Diagnosing prostate cancer	High concentration of apoptotic bodies in lesions	(48)
	B-cell chronic lymphocytic leukemia	Tumor cells	Modifying dendritic cell (DC)-based immunotherapy	Apoptotic bodies act as antigen carriers to allow dendritic cells to process more antigenic information	(54)
	Triple negative breast cancer	Triple negative breast cancer cells	Preventing the proliferation and metastasis of TNBC to the lungs	Inheriting the anti-phagocytic ability and specific homology targeting ability of cancer cells	(57)
	Thrombosis	Mouse B16 melanoma	Leading to thrombosis in cancer patients	fibrin and thrombin production rates are higher in ApoBDs	(55, 56)
Tissue injury and repair	Skin wound healing	Human BMSC	Inducing macrophages to switch to an M2 phenotype	Inhibiting KLF6 by miR-21-5p	(63)
		Adipose tissue-derived stem cell	Balancing the inflammatory polarization state and promoting the healing of chronic wounds.	Activating the JAK-STAT pathway regulated by miR-20a-5p	(65)
		Stem or progenitor	Promoting proliferation and regeneration	The “phoenix rising” pathway	(69)
		Mesenchymal stem cells	Regulating stem cell recruitment and epithelial homeostasis.	Activating the Wnt pathway	(70)
		mesenchymal stem cells	Promoting wound healing and hair regeneration	Activating the Wnt/β-catenin pathway	(72)
	Arteriosclerosis	Smooth muscle cells	Promoting vascular calcification.	Acting as calcium‐concentrating nucleation sites	(77)
		Endothelial progenitor cells	Facilitating the repair of injured endothelium	Promoting proliferation and differentiation	(79)
	Intrauterine adhesions	Bone marrow mesenchymal stem cells	Inhibiting endometrial stromal cell fibrosis	Inhibiting the Wnt/β-catenin signaling pathway	(85)
		Human umbilical cord-derived mesenchymal stem cells	Reducing fibrosis and promote endometrial regeneration	Positive effects on macrophage immunomodulation, cell proliferation, and angiogenesis	(90)
	Osteoporosis	Mesenchymal stem cells	Maintaining stem cell properties	Inhibiting Axin1 by RNF14 and miR-328-3p and activating Wnt/β-catenin pathway	(86)
		Osteoblasts	Inhibiting bone resorption and promoting bone mineralization	Activating RANKL/PI3K/AKT/mTOR/S6K	(92)
	Periodontitis	Osteoclasts	Reducing the loss of alveolar bone	Promoteing Itgb1 expression level by miR-223-3p	(93)
	Osteoarthritis	Macrophages	Alleviating inflammation and inhibiting chondrocyte apoptosis	Inducing the phenotypic switch from M1 to M2 via miR-21-5p.	(94)
	Liver fibrosis	Hepatocyte	Promoting human liver cancer cells	Activating the JAK/STAT-dependent pathway	(98)
		Hepatic stellate cells	Activating the survival pathway in HSC cells and leading to the development of liver fibrosis	Activating PI3K/p38/MAPK/TGF-β/Smad	(99)
		Hepatic stellate cells	survival and diffusion of liver fibrosis	Promoting fibrotic gene expression	(100, 101)
Other diseases	Inflammation of brain	Melanoma cells with high metastatic capacity	Alleviating brain inflammation in a mouse model of Parkinson’s disease (PD)	Loading ASO cross the brain barrier	(107)
	Autoimmune diseases (Type 1 diabetes)	Dendritic cells that engulfed IL-1	Preventive and therapeutic effects on T1D	Inducing autoreactive T cell anergy/apoptosis and stimulates regulatory	(110)
	Systemic lupus erythematosus (SLE)	Somatic cells	Destruction of the ApoBDs clearance process can lead to autoantigen exposure	Translocation of autoantigens into apoptotic bodies	(109)
	Ischemic stroke	plasma neuron and glia	Assessing neurological deterioration and functional outcome in patients with ischemic stroke	There is a correlation between infarct growth and final infarct size	(115)

**Table 2:** Details of the cargo components involved in the function of ApoBDs.

Loaded cargo	Functions	Function in disease	References
RBM11	Promoting the proliferation and drug resistance of surviving Glioblastoma multiforme cells	Disease development	(40)
miRNA-126	Protecting blood vessels in the arteriosclerosis	Treatment of disease	(74)
miRNA-21-5p	Relieving the injury of articular cartilage and the healing of chronic wounds.		(65, 94)
miRNA-20a-5p	Promoting chronic inflammatory wounds healing		(65)
miR-223-3p	Relieving periodontitis		(61)
Wnt8a	Improving wound healing		(66)
miR-328-3p	Maintaining MSC and bone homeostasis		(86)
Vancomycin	Killing Staphylococcus aureus		(111)
doxorubicin (Dox) and indocyanine green (ICG)	killing cells of glioma through the blood-brain barrier		(106)
oligonucleotide (ASO)	Suppressing brain inflammation in mouse models of PD through the blood-brain barrier		(107)
toxic protein saporin and anti-distorting siRNA	Preventing TNBC proliferation and metastasis to the lung		(57)

## 6 Therapeutic strategies and clinical applications of ApoBDs in diseases

**Figure 4:**
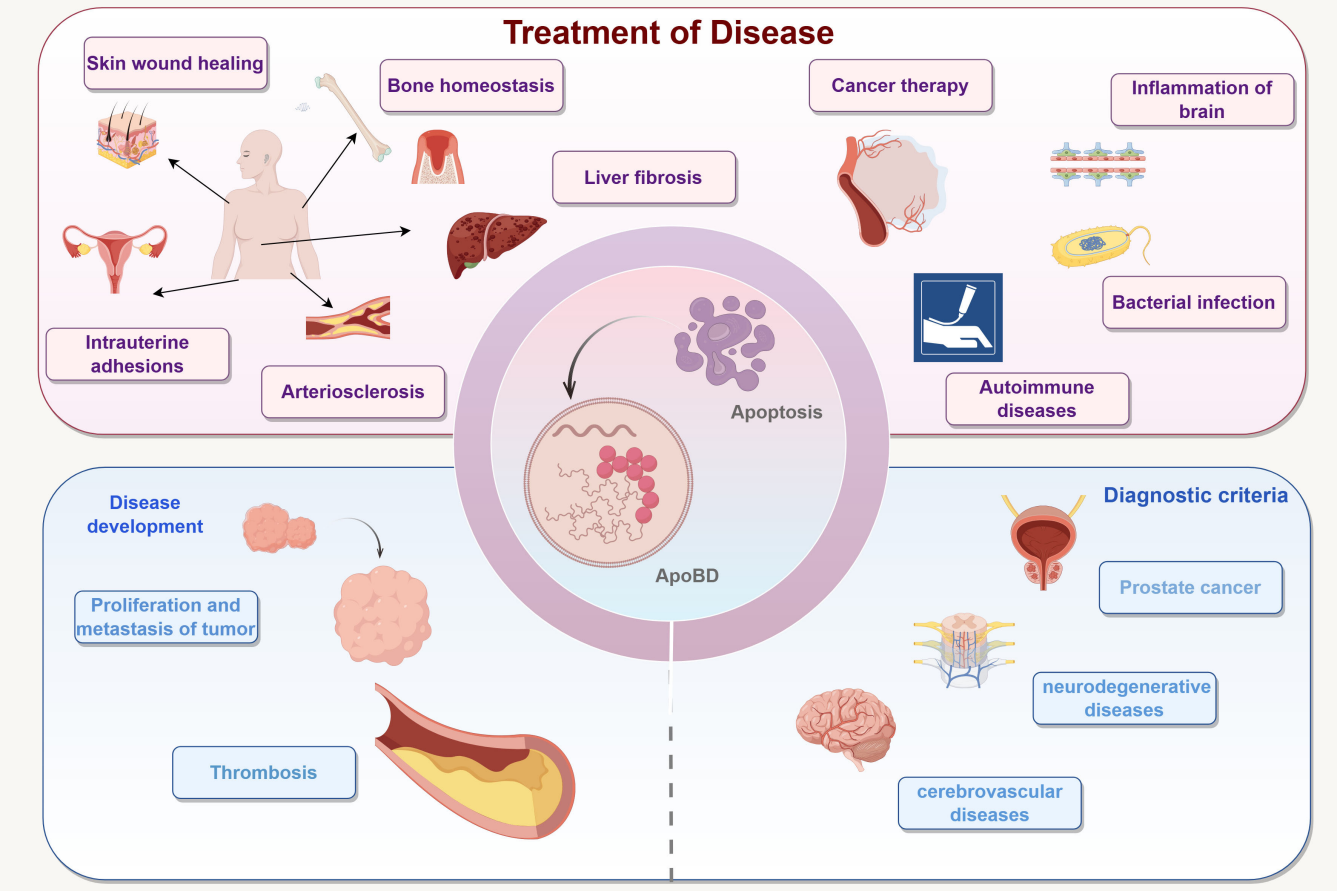
Therapeutic and reparative potential of ApoBDs in disease contexts. Apoptotic bodies exhibit multiple functions in the progression of diseases: they possess both pro-tumorigenic and anti-tumorigenic properties, exert dual regulatory effects during fibrosis, and play a critical role in tissue and organ repair. This figure was created by Figdraw.

ApoBDs, as bioactive extracellular vesicle carriers, have demonstrated promising applications in various fields, including cancer therapy, tissue repair, neurological disorders, and immune regulation (**Figure 4**). Their inherent ability to deliver signaling molecules and antigens to target cells has inspired innovative strategies for targeted drug delivery, vaccine development, and tissue regeneration. Nevertheless, several challenges remain in the application of ApoBDs for tumor therapy. On one hand, ApoBDs derived from different sources exhibit heterogeneity in composition and function; notably, some tumor-derived ApoVs have been found to promote tumor progression, necessitating thorough safety evaluations (40). On the other hand, ApoBDs may be degraded by the lysosomal system after entering recipient cells, thereby reducing their drug-loading efficiency (103). In addition, current isolation and preparation techniques for ApoBDs are still insufficient in completely eliminating irrelevant residues and lack standardized purification protocols, all of which hinder their clinical translation (59, 104, 105).

In the field of tissue repair, ApoBDs derived from stem cells or tissue-specific cells have demonstrated regenerative potential. The *in situ* delivery strategy using MSC-derived ApoBDs highlights the promise of ApoBDs in endometrial regeneration. ApoBDs derived from MSCs or other regenerative cells can be precisely delivered to target cells such as macrophages, fibroblasts, endothelial cells, and stem cells via local injection or incorporation into injectable hydrogel microspheres. These ApoBDs effectively transport bioactive molecules such as miRNAs, mRNAs, proteins, and organelle fragments, thereby promoting the rapid regeneration and repair of various tissues, including skin wounds, osseous and joint tissues, vascular endothelium, and the endometrium. Moreover, through engineering modifications such as ligand-specific targeting and integration with controlled-release carriers, the targeting ability and release kinetics of ApoBDs can be further optimized. This strategy not only circumvents the immunogenicity and tumorigenic risks associated with traditional cell therapies but also offers advantages in large-scale production and personalized customization. Looking ahead, the integration of ApoBDs with physical delivery systems, bioactive molecules, and gene-editing functionalities may fully leverage their dual identity as extracellular vesicles and natural signaling carriers, paving the way for precision regenerative therapies that span from acute injury repair to the reversal of chronic diseases in clinical applications.

In addition to its promising applications in oncology and tissue repair, ApoBDs have demonstrated exceptional drug delivery capabilities across the blood–brain barrier (BBB), offering novel strategies for the targeted treatment of central nervous system (CNS) disorders. Previous studies have successfully loaded doxorubicin (Dox) and indocyanine green (ICG) into ApoBDs, leveraging the selective phagocytosis by macrophages/monocytes to facilitate BBB penetration and achieve precise cytotoxicity against intracranial glioma cells (106). Similarly, ApoBDs derived from highly metastatic melanoma cell lines have been used to deliver antisense oligonucleotides (ASOs) in a mouse model of Parkinson’s disease, effectively suppressing neuroinflammation and improving functional deficits (107). These findings suggest that ApoBDs can serve as biodegradable “micro-shuttles” capable of delivering macromolecular drugs or nucleic acids to pathological sites without compromising BBB integrity. However, the impact of ApoBDs on the BBB varies depending on their cellular origin. For instance, ApoBDs secreted by melanoma cells can disrupt the tight junctions of brain endothelial cells within approximately 60 minutes through transcellular uptake and cytoskeletal rearrangement, thereby weakening the BBB and facilitating tumor invasion and metastasis (108). Therefore, the safety profile of ApoBDs must be rigorously assessed with respect to both their cellular source and cargo.

In the fields of autoimmune diseases and infection control, ApoBDs also demonstrate multiple advantages. In studies on systemic lupus erythematosus (SLE) and type 1 diabetes, autoantigens derived from apoptotic cells are translocated into ApoBDs and subsequently taken up by dendritic cells, leading to the induction of immune tolerance through mechanisms such as the anergy or apoptosis of autoreactive T cells and the expansion of regulatory T cells, thereby alleviating autoimmune pathology (109, 110). For the prevention and treatment of *Staphylococcus aureus* infections, vancomycin-loaded recombinant ApoBDs have exhibited potent bactericidal activity within macrophages and glioblastoma cells, while also enhancing the targeted phagocytosis of pathogens by hepatic and splenic macrophages, resulting in significantly improved anti-infective efficacy and reduced drug-related side effects (111).

ApoBDs not only serve as targeted drug delivery vehicles but also hold potential as biomarkers for disease diagnosis and progression prediction. In cerebrovascular events and neurodegenerative diseases, there is a correlation between the expansion of cerebral infarction and final infarct volume with the quantity of neuron- and glial cell-derived ApoBDs detected in plasma within the first 72 hours post-stroke. The levels of CNS-derived ApoBDs in plasma can be used as predictive markers for neurological deterioration and clinical outcomes in patients with ischemic stroke (112–115). In the future, the dual functions of ApoBDs in multifunctional drug delivery and dynamic disease monitoring are expected to offer a novel paradigm for precision therapy and disease management in central nervous system disorders, autoimmune diseases, and stroke.

In summary, the application advantages of ApoBDs lie in their “cell-free” nature, which circumvents the immunogenicity, potential tumorigenicity, and low transplantation efficiency associated with stem cell therapies. Although existing studies highlight the considerable clinical potential of ApoBDs, their precise therapeutic efficacy and the possibility of eliciting immune responses remain to be validated through clinical practice. Currently, multiple registered randomized controlled trials (RCTs) are systematically evaluating the efficacy and safety of apoptotic vesicles derived from MSCs in various clinical indications, including dry eye, ovarian insufficiency, osteoarthritis, and oocyte maturation (116). Moving forward, further large-scale, multicenter, double-blind RCTs are required to confirm their long-term efficacy and safety. Additionally, standardized protocols for the preparation, characterization, and quantification of apoptotic vesicles must be established, along with identification of key bioactive components and elucidation of their mechanisms of action, to facilitate their translational application in clinical disease diagnosis and treatment.

## 7 Conclusion and prospects

Recent studies on extracellular vesicles have demonstrated that ApoBDs are not merely byproducts of apoptosis, but also possess intercellular communication and cargo delivery functions similar to those of exosomes and microvesicles. With the deepening understanding of the roles of extracellular vesicles, ApoBDs are expected to play unique roles in drug delivery, tissue regeneration, and immunotherapy. ApoBDs derived from diseased cells are no longer regarded solely as “harmful biomolecules” that promote disease progression; rather, their distinct functions offer novel insights for therapeutic strategies.

Despite their therapeutic promise, ApoBDs‐based strategies remain limited by source heterogeneity, unstandardized isolation and purification protocols, unstable and low drug‐loading capacity, potential immunogenicity, and insufficient characterization of optimal administration routes, dosages, and functional variability across different vesicle sources. Therefore, future research could focus on the following areas. (1) designing and optimizing engineered ApoBDs bearing organ- or cell-targeting surface ligands (e.g., peptides or antibodies) for precise delivery. (2) developing high-throughput, standardized isolation protocols—such as combining size-exclusion chromatography with immunoaffinity capture—to ensure batch-to-batch consistency. (3) systematically screening ApoBD cargo to develop robust biomarker assay kits for early disease diagnosis. (4) integrating multimodal imaging reporters into ApoBDs for noninvasive *in vivo* tracking, coupled with pharmacokinetic and pharmacodynamic modeling to refine dosing regimens. Such standardized, integrated strategies will expedite the translation of MSC-derived apoptotic vesicles (including ApoBDs) from preclinical research to routine clinical applications.
